# Supplementary material for: A designed overlapping variant immunogen pool elicits broad sarbecovirus neutralization
Source: bioRxiv. 2026 Jun 4:2026.06.03.729821. Preprint. [Version 1] doi: 10.64898/2026.06.03.729821 (PMC13251928; doi:10.64898/2026.06.03.729821)
Supplement: 1 [file NIHPP2026.06.03.729821v1-supplement-1.pdf]

**Fig.S1. Neutralizing Antibodies are Largely Elicited by the RBD of SARS-CoV-2**

(A-C) Neutralizing antibody titers ($NT_{50}$) against SARS-CoV-$2_{Wu}$ or SARS-CoV-$2_{BA.1}$ pseudotype variants following mock depletion of patient plasma (None) or depletion by the indicated SARS-CoV$2_{Wu}$ Spike(S), NTD(N), RBD(R), or combined NTD and RBD(N+R) proteins. (D-F) Neutralizing antibody titers ($NT_{50}$) against SARS-CoV-$2_{Wu}$ or SARS-CoV-$2_{BA.1}$ pseudotype variants following mock depletion of patient

bioRxiv preprint doi: https://doi.org/10.64898/2026.06.03.729821; this version posted June 4, 2026. The copyright holder for this preprint (which was not certified by peer review) is the author/funder, who has granted bioRxiv a license to display the preprint in perpetuity. It is made available under a CC-BY 4.0 International license.

plasma (None) or depletion by the indicated SARS-CoV2$_{XBB}$ Spike(S), NTD(N), RBD(R), or combined NTD and RBD(N+R) protein. Graph title indicates patient group, each line represents 1 participant, ConVax n=15, Vax3 n=15, Breakthrough n=13. Dotted line indicates the lowest serum dilution tested (1:250).

**Fig.S2. Cross-reactive neutralizing antibodies induced in escape homodimer and heterodimer immunized mice**

(A-D) Neutralizing titers ($NT_{50}$) against SARS-CoV-2 variant pseudotypes in 12 week post-immunization mouse sera following mock depletion (None) or depletion by the indicated RBD protein. Graph title indicates the immunogen used, each line represents1 mouse, n = 4-6 mice per group. Dotted line indicates the lowest serum dilution tested (1:500).

**Fig.S3. Cross-reactive B cells induced in RBD homodimer and heterodimer immunized mice**

(A-D) FACS plots showing mouse lymph node germinal center B cells binding to fluorophore-conjugated RBD$_{Wu}$ and RBD$_{Beta+}$ baits 17 days after footpad immunization with the individual RBD$_{Wu}$-RBD$_{Wu}$ (A) or RBD$_{Beta+}$-RBD$_{Beta+}$ (B) homodimers, a mixture of RBD$_{Wu}$-RBD$_{Wu}$ and RBD$_{Beta+}$-RBD$_{Beta+}$ homodimers (C) or a RBD$_{Wu}$-RBD$_{Beta+}$ heterodimer (D). Numbers indicate the percentage of cells within the gate (that represents binding to both RBD$_{Wu}$ and RBD$_{Beta+}$ monomer baits). Graph title indicates immunogen group. Each plot represents 1 mouse, n=4 mice per group.

**Fig.S4. Neutralizing antibodies elicited by RBD dimers over 12 weeks**

(A-E). Neutralizing titers ($NT_{50}$) over time against SARS-CoV-2$_{Wu}$ and SARS-CoV-2$_{XBB}$ pseudotypes in mouse sera after immunization with two doses (week 0 and week 3) of the indicated immunogens. Graph title indicates immunogen, each line represents 1 mouse, n=6 mice per group. Dotted line indicates the lowest sera dilution tested (1:50).

**Fig.S5. Cross-reactivity of neutralizing antibodies elicited by RBD homodimers, heterodimers and the tiled heterodimer series**

(A-G) Neutralizing titers ($NT_{50}$) against SARS-CoV-2$_{Wu}$ and SARS-CoV-2$_{XBB}$ pseudotypes in 12 week post-immunization mouse sera following mock depletion (None) or depletion by the indicated RBD proteins. Graph title indicates immunogen, each line represents 1 mouse, n = 4-6 mice per group. Dotted line indicates the lowest serum dilution tested (1:500).

**Fig.S6. RBD$_{Wu}$/RBD$_{XBB}$ Cross-binding germinal center B cells elicited by a single RBD$_{Wu}$-RBD$_{XBB}$ heterodimer, mixed RBD$_{Wu}$-RBD$_{Wu}$/RBD$_{XBB}$-RBD$_{XBB}$ homodimers, and the RBD$_{WX5}$ heterodimer pool**

(A–C) FACS plots of mouse lymph node germinal center B cells ($B220^+$, $CD4^-$, $CD8^-$, $NK1.1^-$, $F4/80^-$, $CD38^-$, and $CD95^+$) binding to fluorophore-conjugated monomer RBD baits (RBD$_{Wu}$, RBD$_{XBB}$) for cells harvested on day 17 post-immunization. Bolded numbers represent the percentage of germinal center B cells that bind to both monomeric RBD$_{Wu}$ and RBD$_{XBB}$ baits.

bioRxiv preprint doi: https://doi.org/10.64898/2026.06.03.729821; this version posted June 4, 2026. The copyright holder for this preprint (which was not certified by peer review) is the author/funder, who has granted bioRxiv a license to display the preprint in perpetuity. It is made available under a CC-BY 4.0 International license.

**Fig.S7 $RBD_{Wu}/RBD_{XBB}/RBD_{S1}$ Cross binding germinal center B cells elicited a single $RBD_{Wu}$-$RBD_{XBB}$ heterodimer, mixed $RBD_{Wu}$-$RBD_{Wu}$/$RBD_{XBB}$-$RBD_{XBB}$ homodimers, and the $RBD_{WX5}$ heterodimer pool.** (A–C) FACS plots of Fluorophore conjugated $RBD_{S1}$ binding to mouse lymph node germinal center B cells ($B220^+$, $CD4^-$, $CD8^-$, $NK1.1^-$, $F4/80^-$, $CD38^-$, and $CD95^+$), gated on cells that bound to both $RBD_{Wu}$, and $RBD_{XBB}$. Bolded numbers represent the percentage of $RBD_{Wu}/RBD_{XBB}$ cross reactive B cells that also bind to $RBD_{S1}$.

**Fig.S8. Comparison of neutralizing antibodies elicited by mRNA-LNP or RBD dimer protein immunogens over time**

(A-H) Neutralizing titers ($NT_{50}$) over time against SARS-CoV-$2_{Wu}$ and SARS-CoV-$2_{XBB}$ pseudotypes in mouse sera after immunization with two doses of the indicated immunogens. Graph title indicates immunogen, each line represents 1 mouse, n = 5-6 mice per group. Dotted line indicates the lowest sera dilution tested (1:50).

**Fig.S9. Neutralizing antibodies and virus challenge stocks for evaluation of tiled RBD heterodimer immunogen**

(A,B) Comparison of neutralizing titers ($NT_{50}$) against SARS-CoV-2 variant pseudotypes in mouse sera 12 and 51 weeks post-immunization with two doses of indicated immunogen. Each symbol represents 1 mouse, lines = group mean, n = 5-6 mice per group. Dotted line indicates the lowest sera dilution tested (1:50). (C) Validation of SARS-CoV-2 challenge virus stocks, KP.3 and KP.3.1. Lung viral loads (SARS-CoV-2 RNA copies per µg total RNA) on day 3 after infection of K18-hACE2 mice. Each symbol represents 1 mouse, lines = group geometric mean, n=5 mice per group (D) Lung viral loads (rVSV/SARS-1 RNA copies per µg total lung RNA) on each of the indicated hours after infection of K18-hACE2 IFNAR(-/-) mice. Each symbol represents 1 mouse, dotted line=limit of detection.

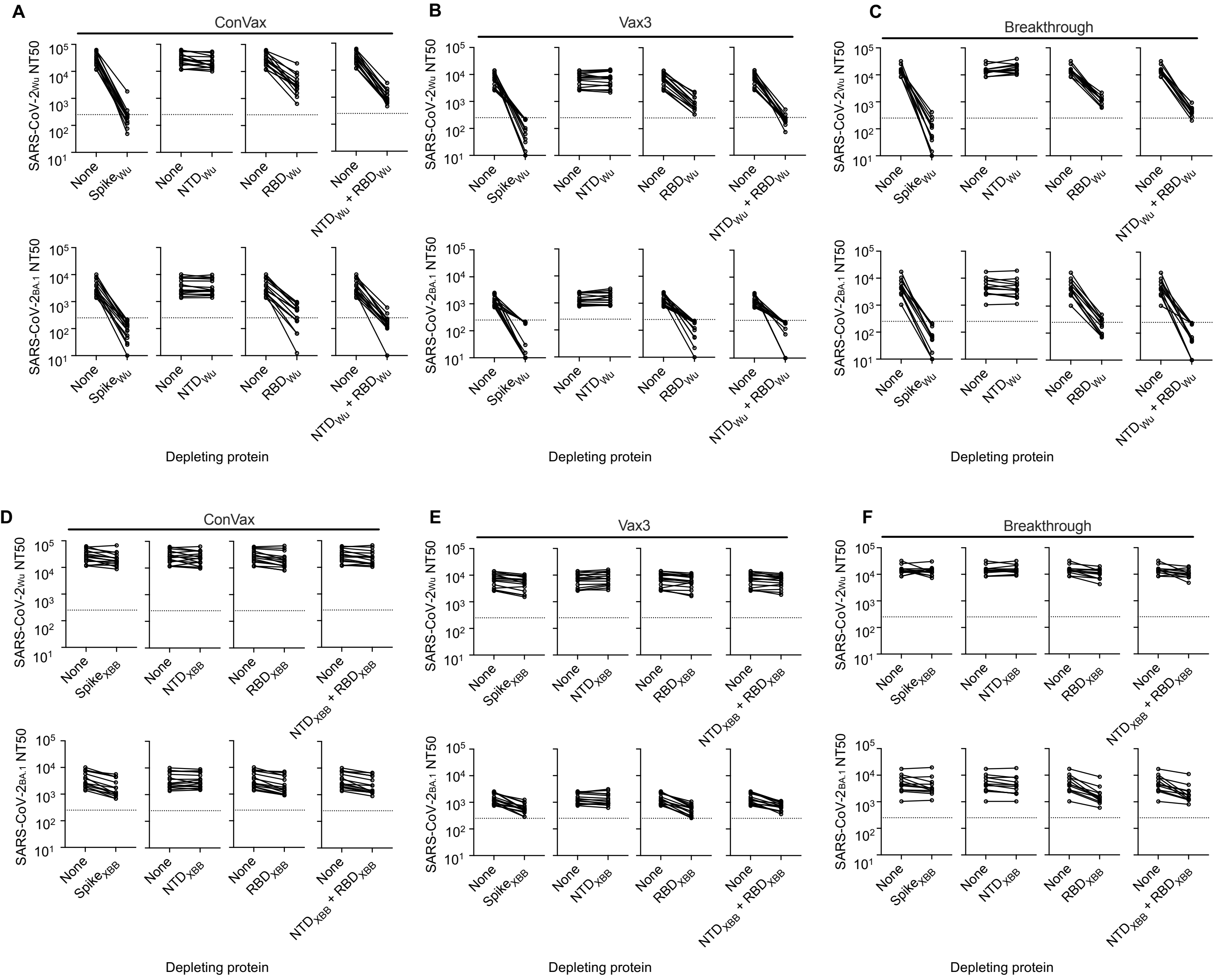

A
ConVax
B
Vax3
C
Breakthrough
D
ConVax
E
Vax3
F
Breakthrough
SARS-CoV-2Wu NT50
SARS-CoV-2BA.1 NT50
10^1
10^2
10^3
10^4
10^5
None
SpikeWu
NTDWu
RBDWu
NTDWu + RBDWu
SpikeXBB
NTDXBB
RBDXBB
NTDXBB + RBDXBB
Depleting protein


Figure S1

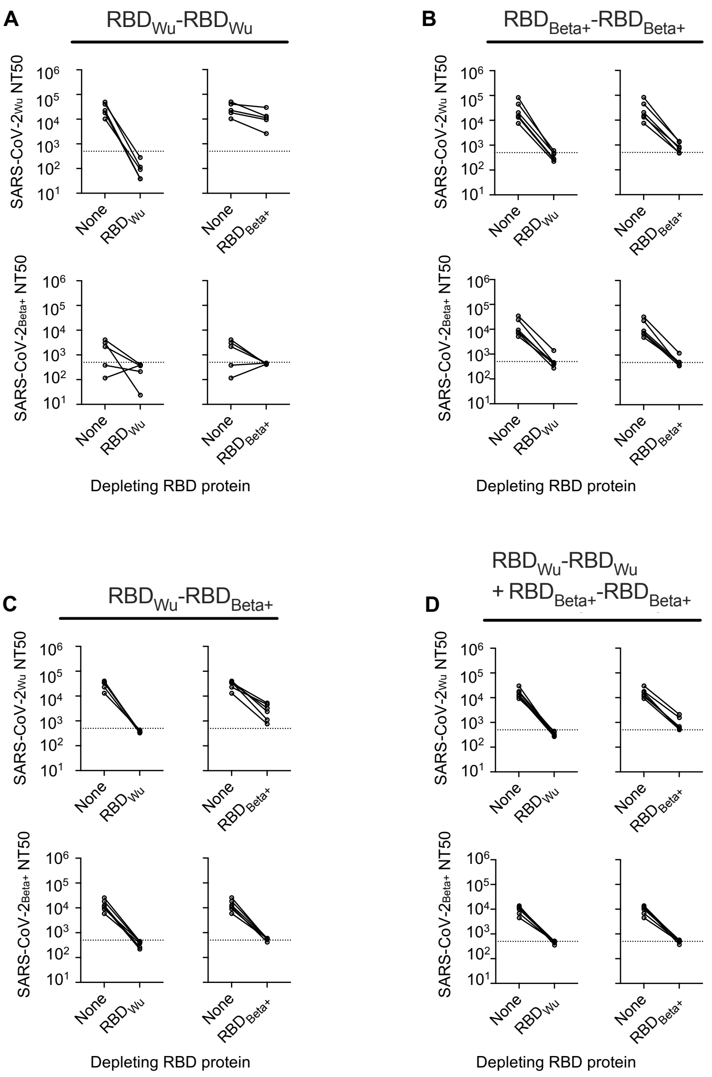
A
RBDWu-RBDWu
B
RBDBeta+-RBDBeta+
C
RBDWu-RBDBeta+
D
RBDWu-RBDWu
+ RBDBeta+-RBDBeta+
SARS-CoV-2Wu NT50
SARS-CoV-2Beta+ NT50
None
RBDWu
RBDBeta+
Depleting RBD protein

Figure S2

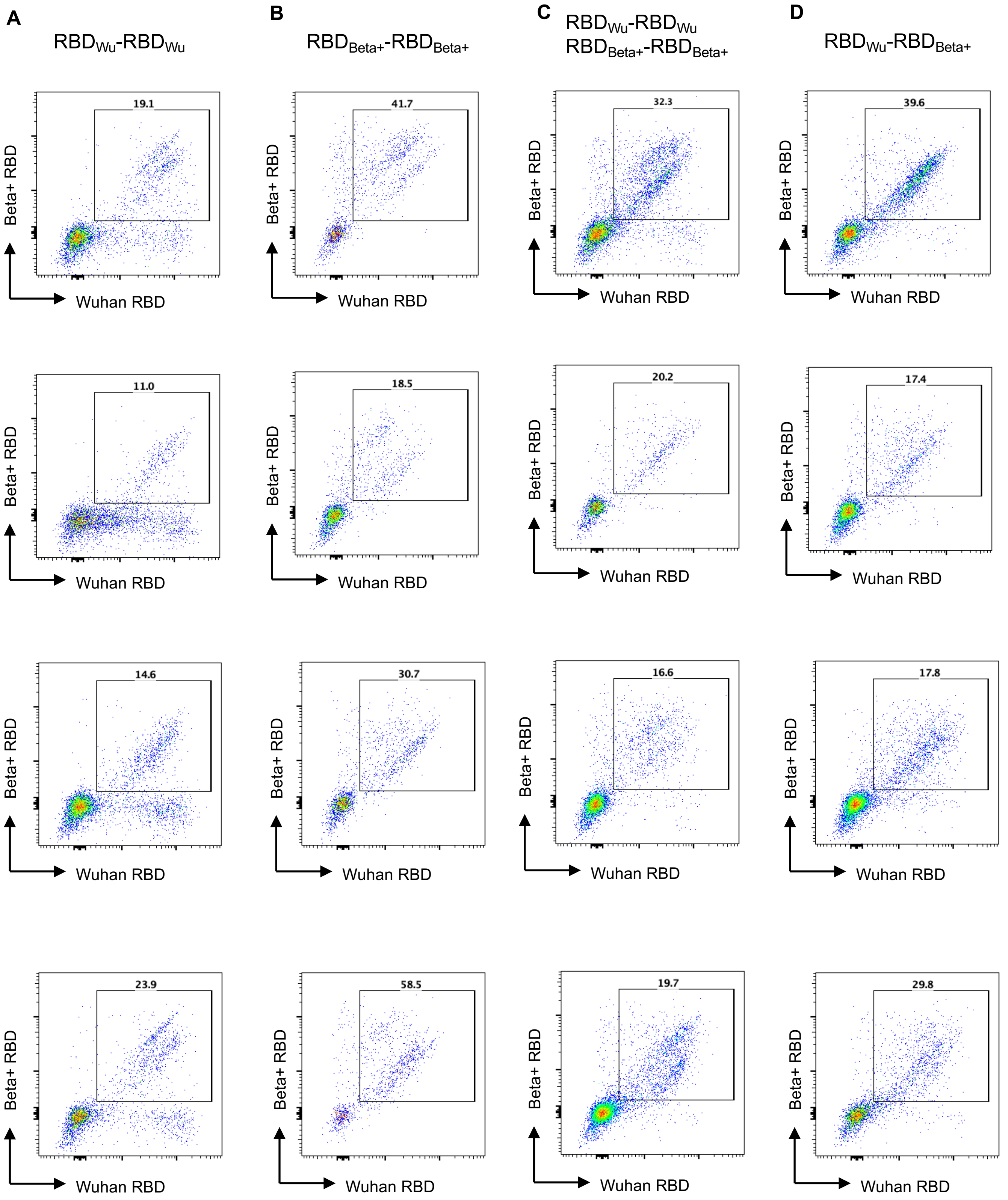
A
RBDWu-RBDWu
B
RBDBeta+-RBDBeta+
C
RBDWu-RBDWu
RBDBeta+-RBDBeta+
D
RBDWu-RBDBeta+
19.1
41.7
32.3
39.6
11.0
18.5
20.2
17.4
14.6
30.7
16.6
17.8
23.9
58.5
19.7
29.8
Beta+ RBD
Wuhan RBD

Figure S3

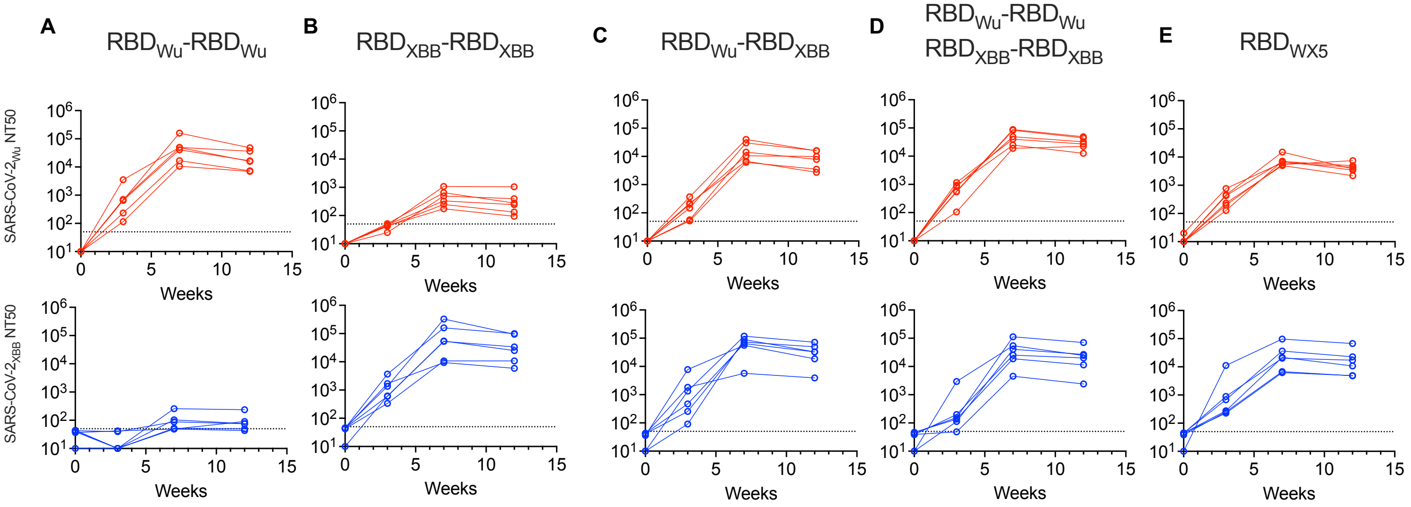

A
$RBD_{Wu}$-$RBD_{Wu}$
B
$RBD_{XBB}$-$RBD_{XBB}$
C
$RBD_{Wu}$-$RBD_{XBB}$
D
$RBD_{Wu}$-$RBD_{Wu}$
$RBD_{XBB}$-$RBD_{XBB}$
E
$RBD_{WX5}$
SARS-CoV-$2_{Wu}$ NT50
SARS-CoV-$2_{XBB}$ NT50
Weeks


Figure S4

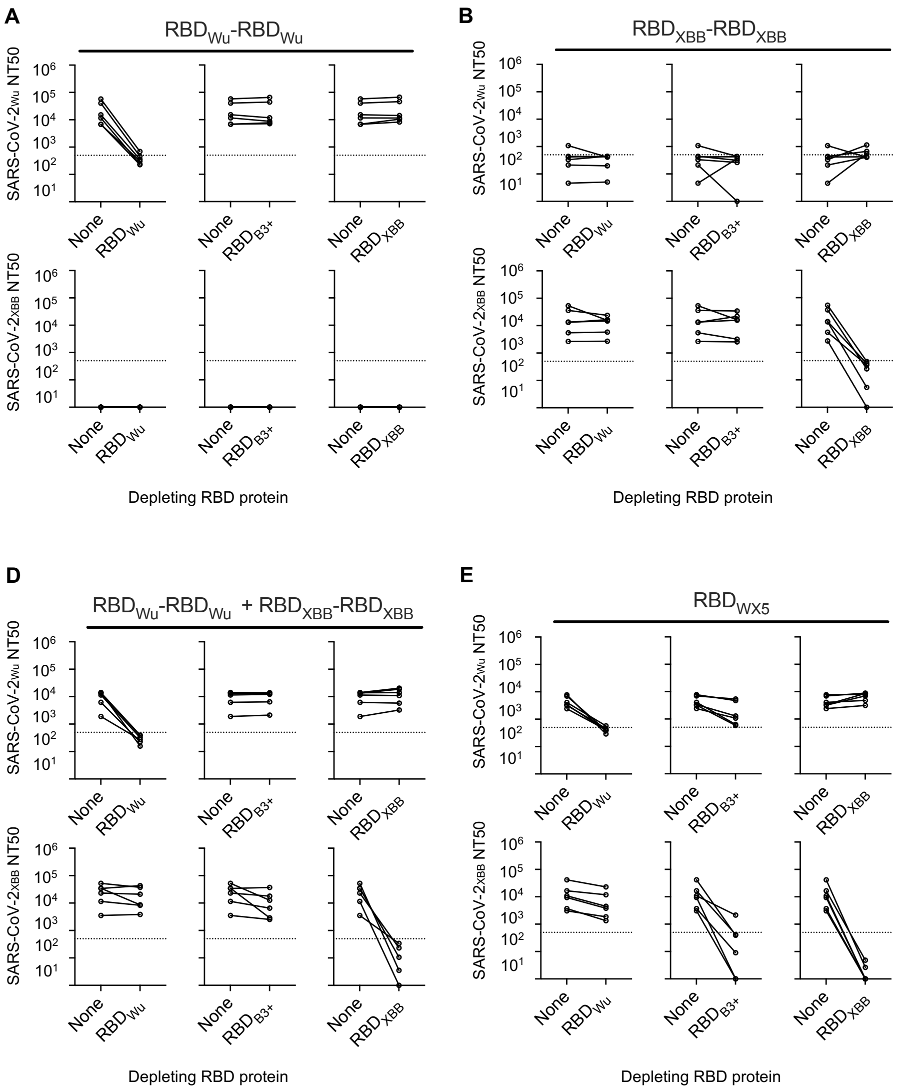
A
RBDWu-RBDWu
B
RBDXBB-RBDXBB
C
RBDWu-RBDXBB
D
RBDWu-RBDWu + RBDXBB-RBDXBB
E
RBDWX5
SARS-CoV-2Wu NT50
SARS-CoV-2XBB NT50
None
RBDWu
RBDB3+
RBDXBB
Depleting RBD protein

Figure S5

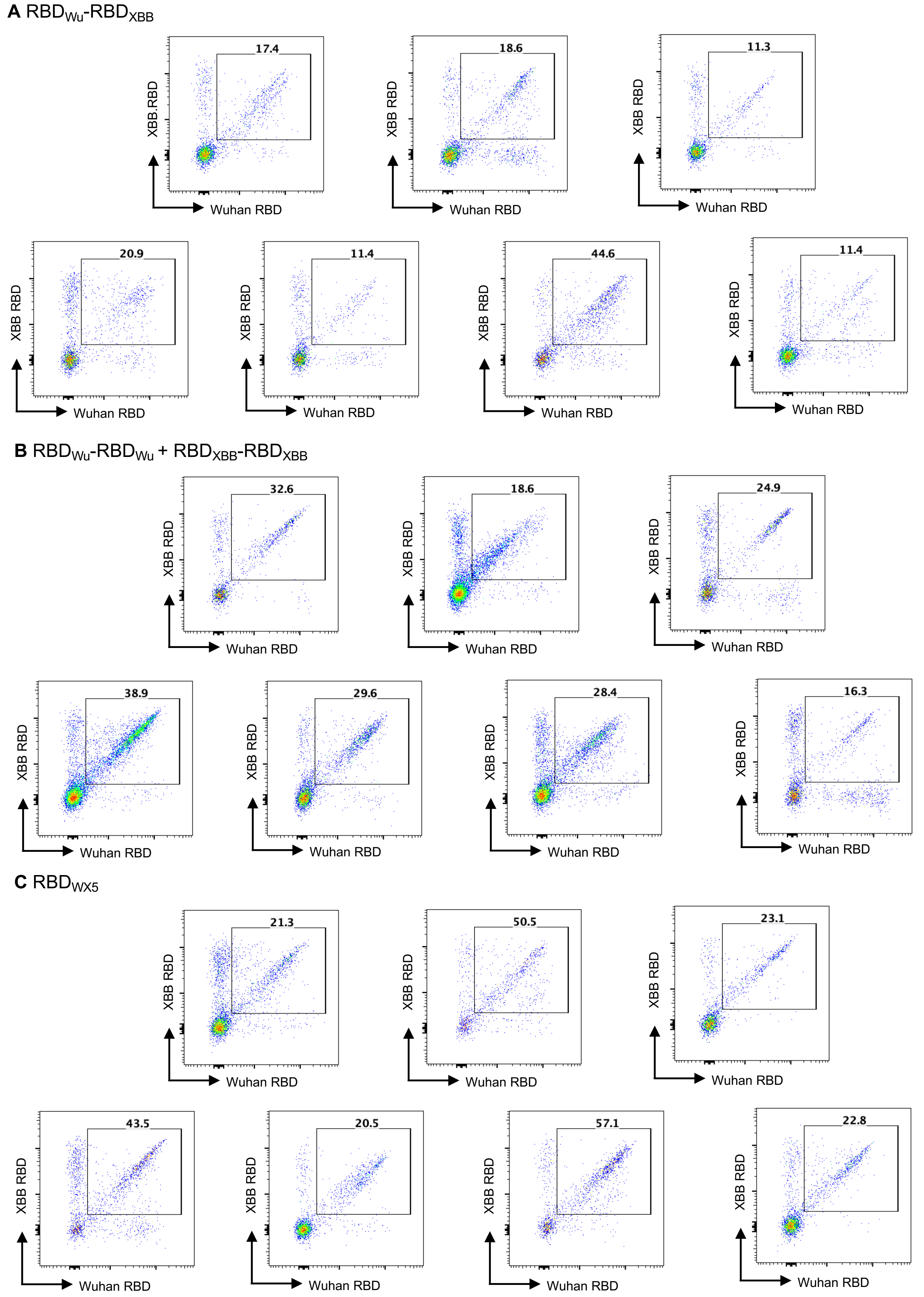
A RBDWu-RBDXBB
17.4
18.6
11.3
20.9
11.4
44.6
11.4
B RBDWu-RBDWu + RBDXBB-RBDXBB
32.6
18.6
24.9
38.9
29.6
28.4
16.3
C RBDWX5
21.3
50.5
23.1
43.5
20.5
57.1
22.8
XBB RBD
Wuhan RBD

Figure S6

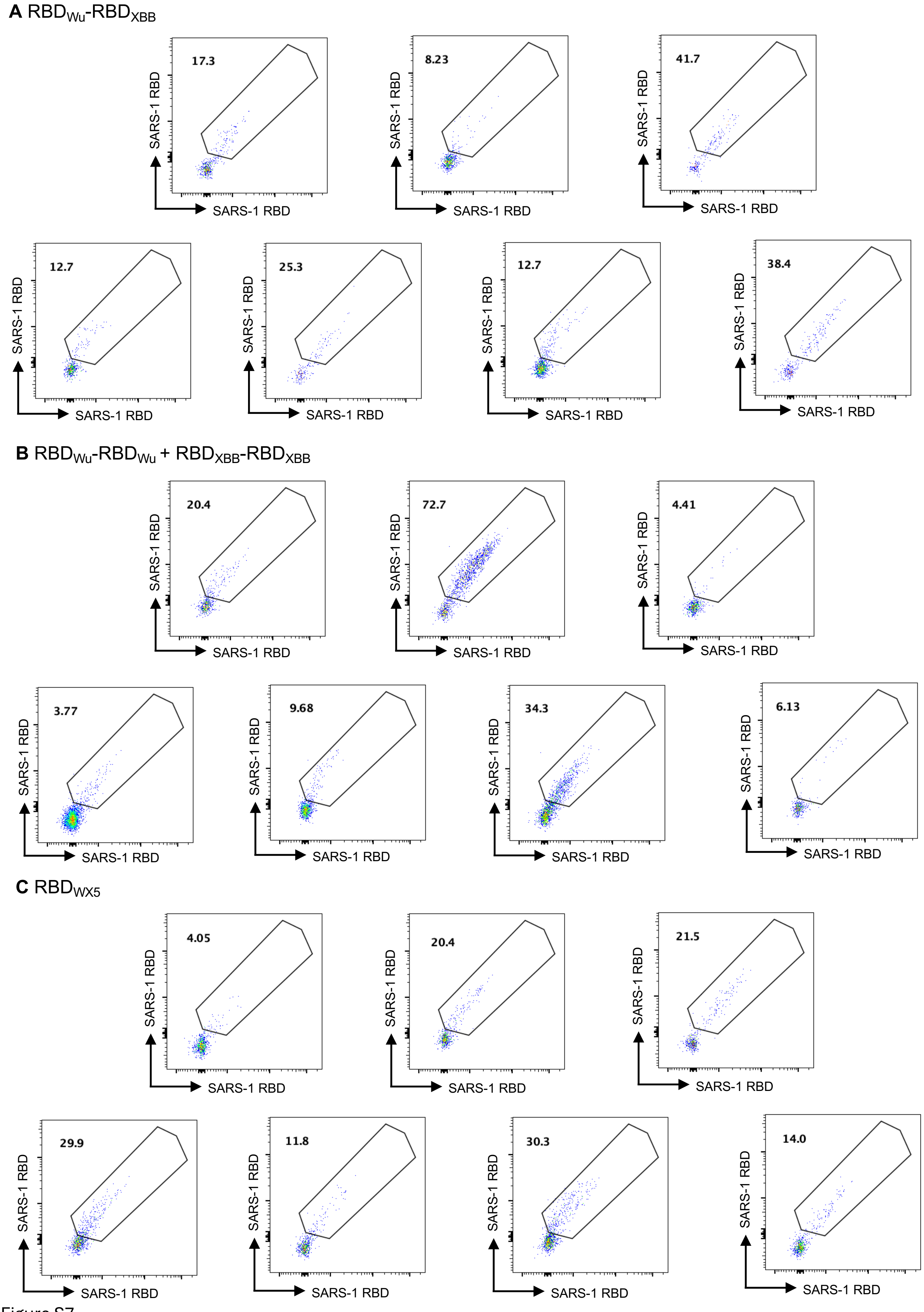
A RBDWu-RBDXBB
17.3
8.23
41.7
12.7
25.3
12.7
38.4
B RBDWu-RBDWu + RBDXBB-RBDXBB
20.4
72.7
4.41
3.77
9.68
34.3
6.13
C RBDWX5
4.05
20.4
21.5
29.9
11.8
30.3
14.0
SARS-1 RBD

Figure S7

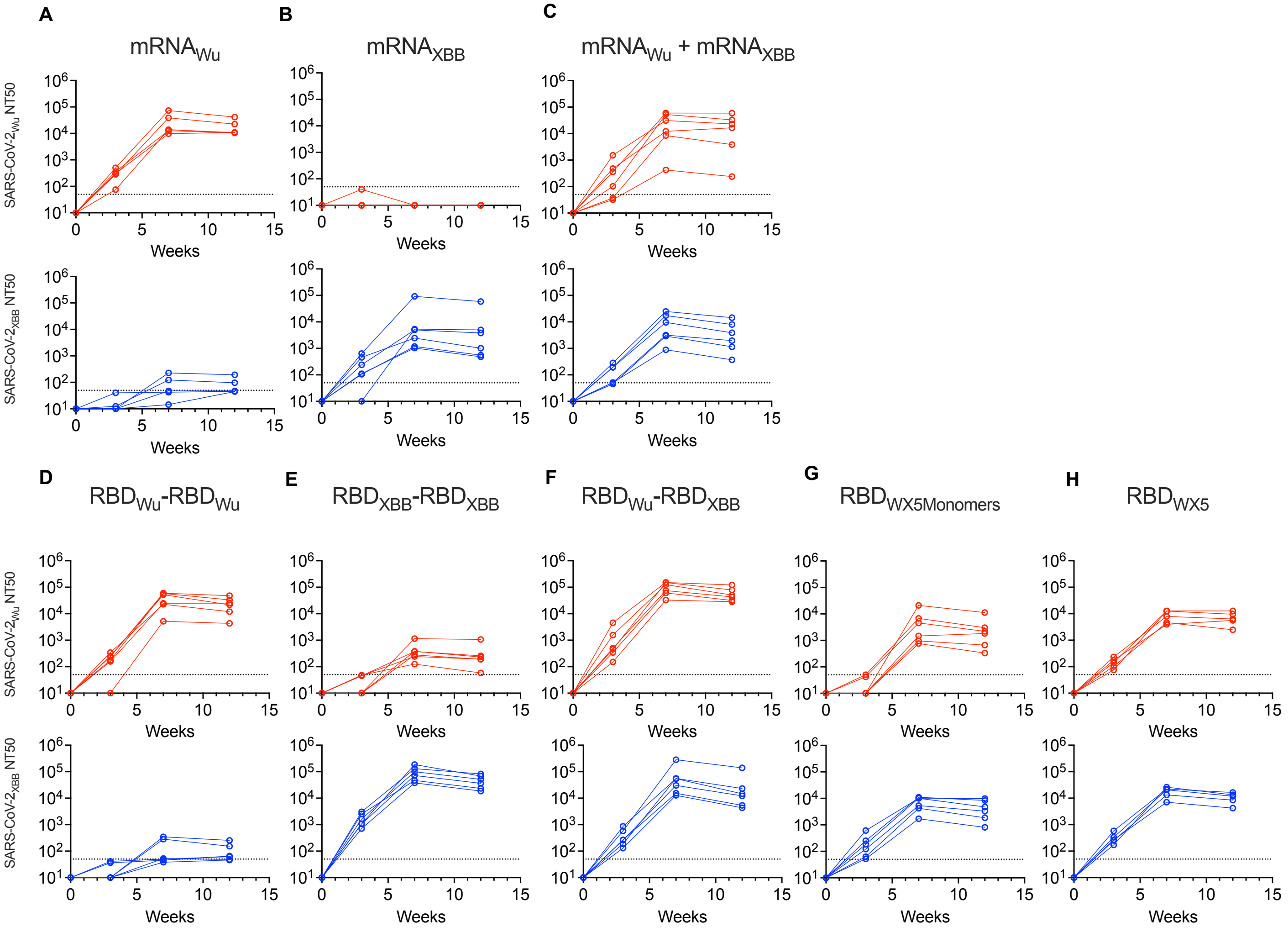

A
mRNA_Wu
B
mRNA_XBB
C
mRNA_Wu + mRNA_XBB
SARS-CoV-2_Wu NT50
SARS-CoV-2_XBB NT50
Weeks
D
RBD_Wu-RBD_Wu
E
RBD_XBB-RBD_XBB
F
RBD_Wu-RBD_XBB
G
RBD_WX5Monomers
H
RBD_WX5


Figure S8

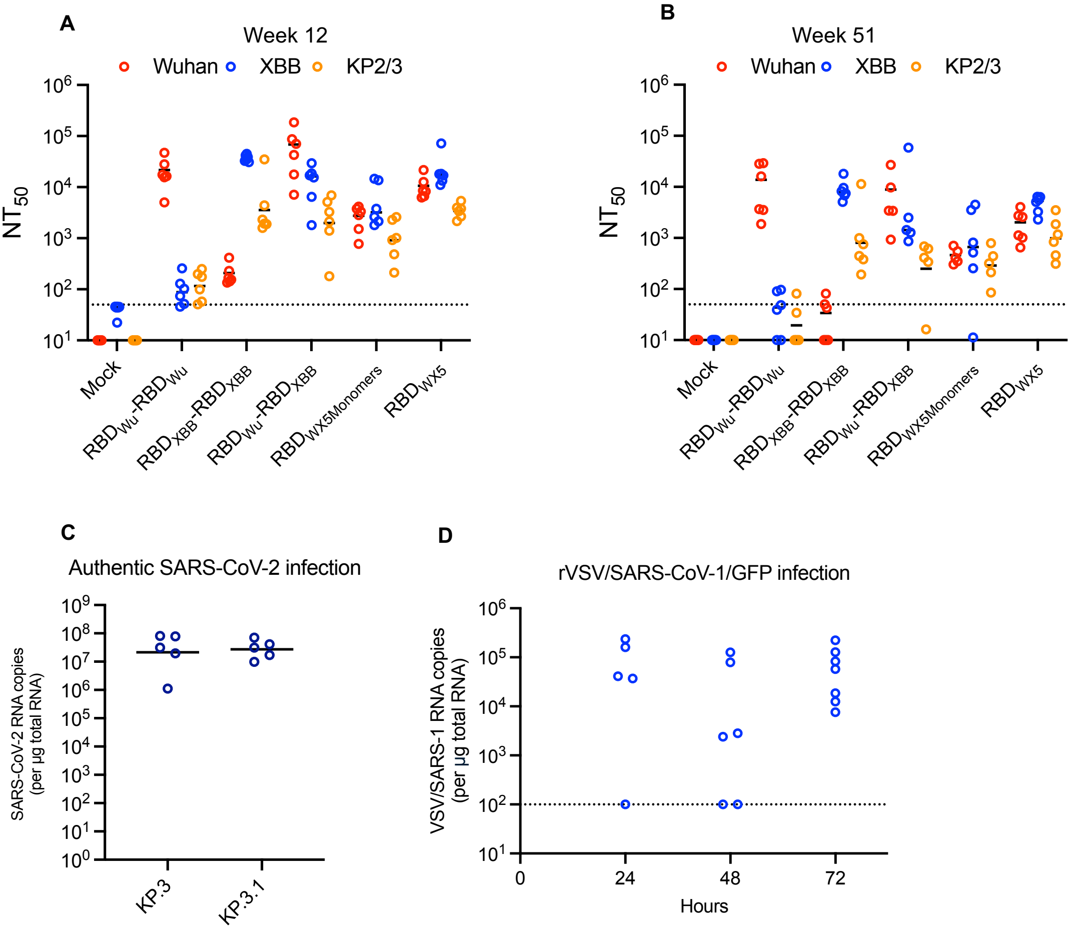
A
Week 12
Wuhan
XBB
KP2/3
NT50
Mock
RBDWu-RBDWu
RBDXBB-RBDXBB
RBDWu-RBDXBB
RBDWX5Monomers
RBDWX5
B
Week 51
C
Authentic SARS-CoV-2 infection
SARS-CoV-2 RNA copies (per µg total RNA)
KP.3
KP.3.1
D
rVSV/SARS-CoV-1/GFP infection
VSV/SARS-1 RNA copies (per µg total RNA)
0
24
48
72
Hours

Figure S9
